# Research on the Absorption Properties of Fe_70_Ni_30_ Alloy/SiO_2_ Coated Continuous Glass Fiber Composites by Magnetron Sputtering

**DOI:** 10.3390/ma19122552

**Published:** 2026-06-12

**Authors:** Zhuohui Zhou, Mengyu Zhou, Zhiyong Wang, Yan Zhao

**Affiliations:** 1 School of Materials Science and Engineering, Beihang University, Beijing 100095, China; zhouzhuohui@126.com; 2 AECC Beijing Institute of Aeronautical Materials, Beijing 100095, China

**Keywords:** microwave absorption materials, composite materials, magnetron sputtering, continuous glass fiber reinforcement materials

## Abstract

In this study, Fe_70_Ni_30_ metal was deposited onto continuous glass fiber composites via magnetron sputtering, followed by surface coating with SiO_2_. The effects of key process parameters-including Fe_70_Ni_30_ sputtering duration (2, 5, 10, 20, and 30 min) and SiO_2_ surface coating-on the electromagnetic properties and microwave absorption performance of the materials were systematically investigated. Scanning electron microscopy (SEM) characterization revealed that as sputtering time increased, the metal coating evolved from discrete small particles into a continuous film. Cross-sectional SEM analysis further demonstrated the formation of a bilayer structure after SiO_2_ introduction. X-ray diffraction (XRD) patterns confirmed the presence of diffraction peaks corresponding to the Fe_70_Ni_30_ alloy solid solution. Electromagnetic parameter measurements indicated that the influence of sputtering time on electromagnetic properties was primarily pronounced during the metal layer growth stage; once a continuous film was formed, the variation in electromagnetic parameters diminished. Concurrently, the SiO_2_ coating exhibited a significant regulatory effect on dielectric parameters. Reflection coefficient calculations showed that the optimal absorption thickness for the single-layer material ranged from 2.5 to 3.0 mm, with the absorption peak shifting toward lower frequencies as thickness increased. However, the effective absorption bandwidth (EAB) was only 3–5 GHz, failing to meet wideband requirements. In contrast, the three-layer composite structure (total thickness: 3.8 mm) optimized via genetic algorithm achieved impedance gradient and loss synergy, expanding the EBW (R < −10 dB) from 4.8 GHz (single layer) to 10 GHz (8–18.0 GHz)-a substantial improvement over the single-layer configuration. This work provides experimental evidence and technical support for the structural design and process optimization of lightweight, high-efficiency, wideband microwave-absorbing materials.

## 1. Introduction

With the rapid advancement of radar detection technology and modern electronic warfare, the demand for electromagnetic wave-absorbing materials has grown increasingly pronounced across fields such as military stealth, electromagnetic shielding, and human protection [1,2,3,4,5]. An ideal wave-absorbing material is expected to integrate the core attributes of “thinness, light weight, broad bandwidth, and high efficiency”-namely, achieving wideband and high-performance absorption within a lightweight structure while satisfying mechanical load-bearing requirements [6,7,8].

Continuous glass fiber-reinforced composites (GFRPs), renowned for their exceptional mechanical properties, corrosion resistance, and design flexibility, have emerged as a focal point in research on structurally integrated functional wave-absorbing materials [9,10,11]. However, pristine glass fibers exhibit a “wave-transparent” behavior toward electromagnetic waves, necessitating the construction of materials with electromagnetic loss capabilities on their surfaces to enable effective wave absorption [12].

Magnetron sputtering deposition technology exhibits distinct advantages in the fabrication of functional materials, attributed to its controllable deposition rate, strong film-substrate adhesion, precisely tunable composition, and environmental benignity [13]. In recent years, researchers have successfully prepared diverse composite materials on various substrates via magnetron sputtering. Kang et al. [14] investigated the magnetron sputtering synthesis of graphene/copper composite systems and their multispectral stealth performance, based on the design of an electromagnetic cage structure. Prokhorenkova et al. [15] fabricated Co-C microwave-absorbing coatings through magnetron sputtering and systematically analyzed their structural and mechanical properties. For fiber fabric substrates, Wang et al. [16] constructed Ni-Cr alloy wave-absorbing structures on the surface of fiber fabrics via magnetron sputtering, verifying the feasibility of this process for depositing uniform metal coatings on flexible substrates. Nam et al. [17] developed lightweight thin-layer radar-absorbing structures by sputtering silver layers. Furthermore, magnetron sputtering has been extensively employed in the design of composite coatings, including graphene/silicon carbide multilayer structures, magnesium alloy composite low-absorption coatings, and black films for optical devices [18]. These applications highlight the flexibility and controllability of this technology in regulating multilayer film structures.

In the design of electromagnetic wave-absorbing material systems, Fe_70_Ni_30_ alloy-a typical soft magnetic material-has garnered significant attention in the field of electromagnetic wave absorption due to its combination of high saturation magnetization and excellent magnetic permeability. Studies have demonstrated that Fe_70_Ni_30_ exhibits superior magnetic properties; its composite with carbon materials [19], carbon foams [20], and other substrates can effectively optimize impedance matching characteristics and attenuation capabilities.

However, single metal coatings often suffer from poor impedance matching and high surface reflectivity of electromagnetic waves, which limits further improvements in their absorption performance. Surface modification via the introduction of a dielectric layer is an effective strategy to address this issue. As a low-dielectric-constant ceramic material, SiO_2_ not only serves as an impedance matching layer to regulate the surface electromagnetic parameters of the coating but also enhances the oxidation resistance and environmental stability of the composite. For instance, Zou et al. [20] significantly improved the low-frequency absorption performance of FeNi-alloy-coated carbon foams by introducing a SiO_2_ layer on their surface. Guo, Q., et al. [21] designed a hollow conductive magnetic FeNi@SiO_2_@polypyrrole (PPy) (FNSP) nanorod. Result shows that the SiO_2_ intermediate layer further improved interfacial polarization and multi-reflection and scattering behaviors, with a minimum reflection loss (RLmin) of −76.38 dB at a thickness of 1.47 mm and an effective absorption bandwidth (EAB) of 5.42 GHz at 1.73 mm. Zou, Y., et al. [22] used the magnetron sputtering method to deposit a series of nano-granular films with different SiO_2_ contents onto carbon foam, creating hierarchical CMF/(FeNi)x(SiO_2_)1−x composites. When the SiO_2_ content in the granular film is 5%, the CMF/(FeNi)_0.95_(SiO_2_)_0.05_ sample exhibits the best absorption performance, achieving a minimum reflection loss of −56.3 dB at 2.5 mm and a maximum effective absorption bandwidth of 8 GHz at 2.7 mm.

Despite extensive research on magnetron sputtering for functional coating fabrication and FeNi alloy-based wave-absorbing materials, systematic studies on depositing Fe_70_Ni_30_ alloy coatings onto continuous glass fibers via magnetron sputtering, followed by surface modification with a SiO_2_ layer, remain scarce in the literature. Building on this research gap, this study fabricates Fe_70_Ni_30_ metallic coatings on continuous glass fiber composites using magnetron sputtering and introduces a SiO_2_ layer for surface encapsulation of the metallic coatings. The effects of varying sputtering parameters on the coating microstructure and electromagnetic properties are systematically investigated, and the enhancement mechanism of the SiO_2_ modification layer on the wave-absorbing performance of the composites is explored. This work aims to provide theoretical insights and experimental support for the design and fabrication of high-performance structural wave-absorbing composites.

## 2. Experiment

### Materials

The primary raw material was glass fiber cloth (grade: EW100A-100a), a plain-weave fabric with a warp and weft fiber density of 20 fibers per cm, supplied by the Nanjing Institute of Glass Fiber Research (Nanjing, China). The density of the fiber cloth is 100 g/m^2^. Fe_70_Ni_30_ and SiO_2_ targets were procured from Zhongnuo New Material Co., Ltd. (Beijing, China), with a size of Φ50.8 mm and a purity of 99.9%. The resin employed was a medium-temperature epoxy resin with a density of 1.2 g/cm^3^, provided by Zhengzhou University (Zhengzhou, China). Acetone and deionized water were used as auxiliary materials for cleaning purposes.

## 3. Experimental Procedure

### 3.1. Experimental Preparation

Glass cloth was cut into dimensions of 330 mm × 120 mm, and then immersed in a 1:1 mass ratio mixture of acetone and deionized water for ultrasonic cleaning for 20 min. During sonication, the cloth was gently agitated occasionally with a glass rod to remove surface impurities such as organic solvents and dust. After ultrasonic treatment, the cloth was rinsed thoroughly with deionized water three times, and then dried in an oven at 80 °C for 15 min. The dried samples were sealed in sample bags and stored in a desiccator for subsequent use.

### 3.2. Preparation of Fiber Cloth Coated Samples

**Figure 1:**
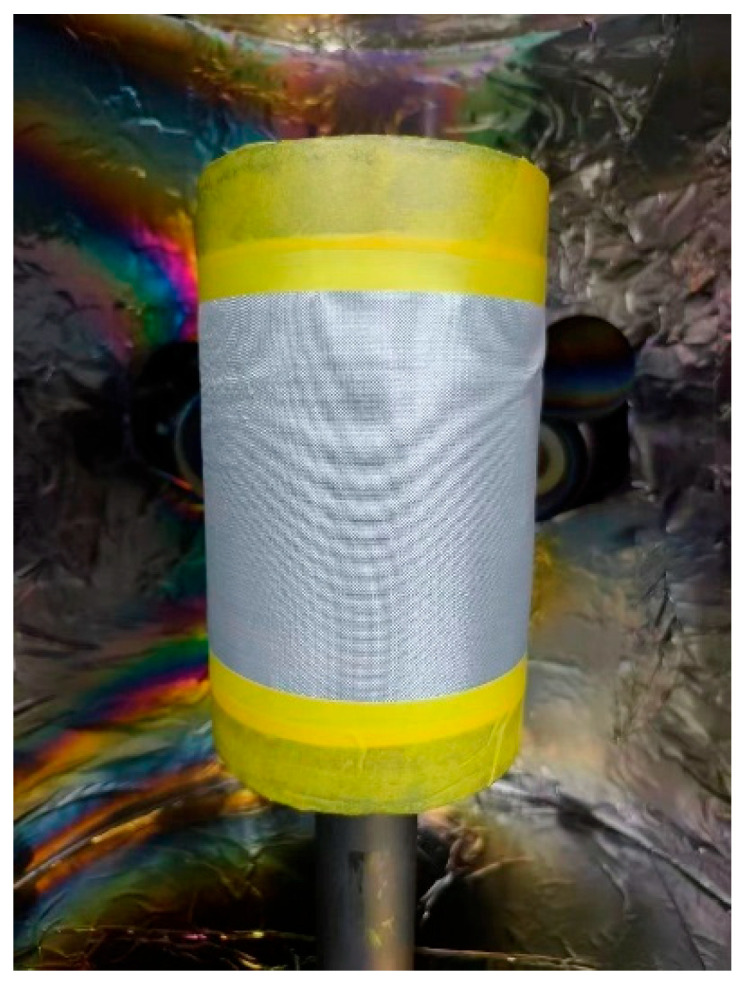
Schematic of the magnetron sputtering experimental setup.

A magnetron sputtering system was employed, where the glass fiber cloth was laid flat on a cylindrical substrate and secured at the edges with adhesive tape (Figure 1). The distance between the glass fiber cloth and the target was set to 100 mm, with a base vacuum of 1.0 × 10^−3^ Pa and a sputtering power of 0.5 kW. The sputtering pressure was maintained at 0.5 Pa, and the substrate rotation speed was controlled at 5 rpm to ensure uniform film deposition. Two sets of samples were fabricated by adjusting the sputtering duration:

Set 1: deposited using a direct current (DC) power supply with an Fe_70_Ni_30_ alloy target.

Set 2: first deposited with an Fe_70_Ni_30_ alloy layer via DC sputtering, followed by a SiO_2_ encapsulation layer via radio frequency (RF) sputtering (fixed sputtering time of 10 min and sputtering power of 0.2 kW for the SiO_2_ layer).

The varying process parameters are summarized in Table 1.

### 3.3. Preparation of Prepreg

Epoxy resin and acetone were mixed uniformly at a mass ratio of 1:1 to form a resin solution. The solution was spray-coated onto all coated fiber cloths using a spray gun with a nozzle diameter of 0.5 mm and an operating pressure of 10~30 kPa. The coating weight per pass was controlled at approximately 10~15 g/m^2^. Subsequent coats were applied only after the previous layer had air-dried to a touch-dry state. This process was repeated until the areal density of the prepreg increased by 70 ± 5 g/m^2^, after which the prepreg was set aside for subsequent use.

### 3.4. Preparation of Laminates

The resin-impregnated coated fiber cloths were laid up in the [0]_20_ stacking sequence, sealed in a vacuum bag, and then transferred to an autoclave for curing. The curing cycle was as follows:

Vacuum application: the system was evacuated to a vacuum level of ≤−0.0095 MPa;

Pressure and temperature ramp-up: pressurization was initiated at room temperature at a rate of 0.03 MPa/min to a target pressure of 0.3 MPa, with simultaneous heating at a rate of 1.5 °C/min;

First holding stage: the temperature was held at 80 °C for 30 min;

Second heating ramp: the temperature was further increased at 1 °C/min to 135 °C;

Second holding stage: the temperature was held at 135 °C for 120 min;

Cooling stage: the system was cooled under pressure at a rate of 1 °C/min to 60 °C, after which the laminate was removed from the autoclave.

## 4. Results and Discussion

### 4.1. SEM Morphology Analysis

**Figure 2:**
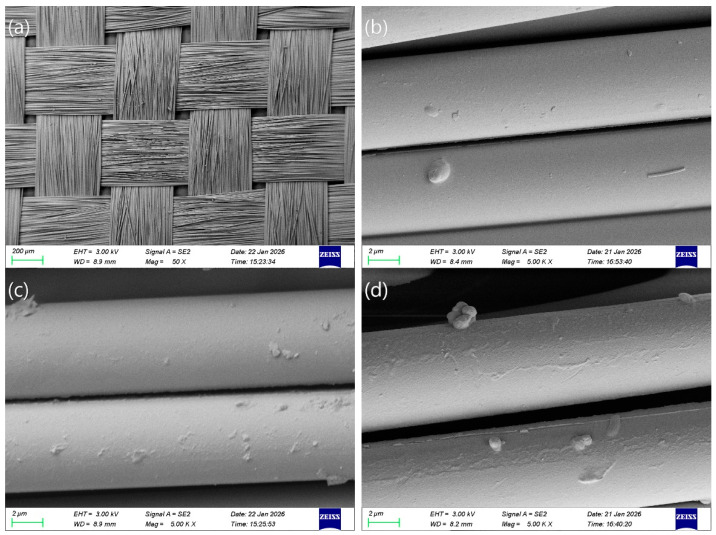

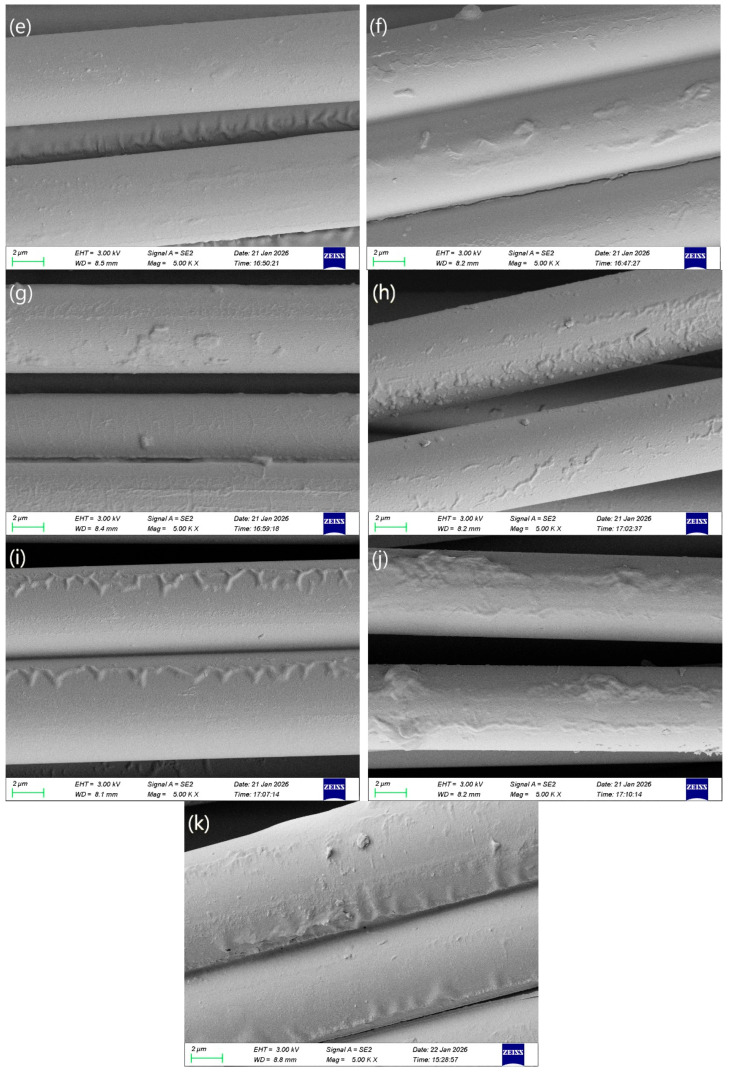
(**a**) The Fiber Cloth and Distribution of Coatings on Fiber Surfaces Under Different Coating Processes; (**b**) 2 min FeNi; (**c**) 5 min FeNi; (**d**) 10 min FeNi; (**e**) 20 min FeNi; (**f**) 30 min FeNi; (**g**) 2 min FeNi + SiO_2_; (**h**) 5 min FeNi + SiO_2_; (**i**) 10 min FeNi + SiO_2_; (**j**) 20 min FeNi + SiO_2_; (**k**) 30 min FeNi + SiO_2_.

The scanning electron microscope (SEM) employed was a JEOL JSM-7500F (JEOL Ltd., Tokyo, Japan), operated at accelerating voltages ranging from 0.1 to 30 kV and capable of magnifications from 25× to 200,000×. Figure 2 presents the SEM characterization results of the coated fiber fabrics. Specifically, Figure 2a illustrates the weave structure of the fiber cloth, which is identified as a plain weave interlaced by fiber bundles oriented at ±90°. Figure 2b–f display the surface morphologies of fiber filaments from Samples 1#–5#. Under the process condition of shorter sputtering time, the coatings initially exhibit a granular distribution (Samples 1#–3#). With increasing sputtering time, the granular coatings gradually coalesce into a continuous film (Sample 4#), and in some regions, the coating even bridges the gaps between adjacent fiber filaments, forming inter-filament connections (Sample 5#). Figure 2g–k show the surface states of fiber filaments from Samples 6#–10#. Notably, the coating on Sample 6# has essentially formed a continuous film, which is attributed to the longer sputtering duration for SiO_2_. This observation confirms that the SiO_2_ coating can fully encapsulate the underlying metal coating. Additionally, the particulate features on the sample surfaces reflect the growth mechanism of the metal coating. Sample 10# also exhibits inter-filament coating connections between some adjacent fiber filaments.

**Figure 3:**
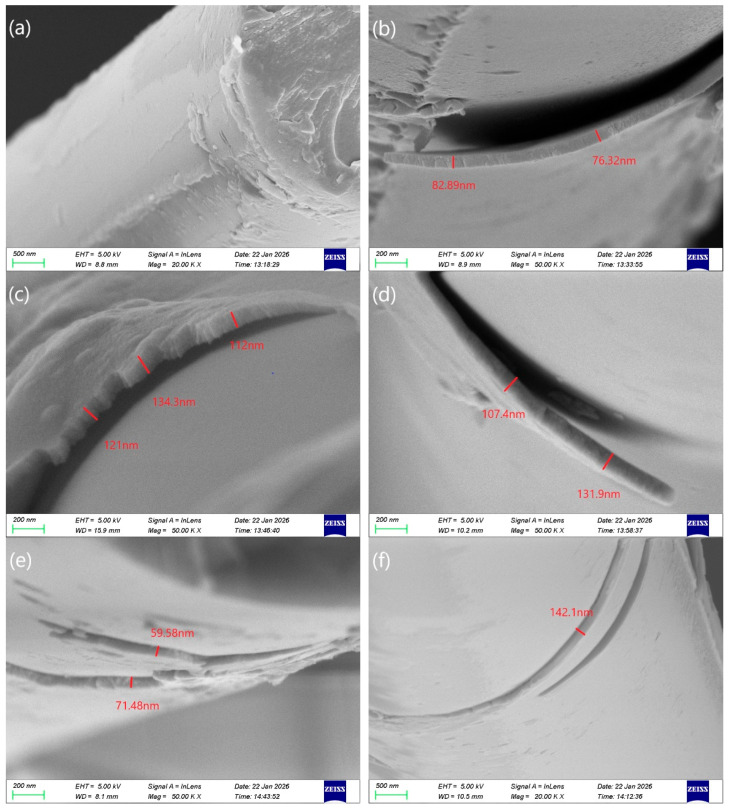
Cross-Sectional Morphologies of Coatings for Different Samples: (**a**) 3#, (**b**) 4#, (**c**) 5#, (**d**) 8#, (**e**) 9#, (**f**) 10#.

To further characterize the coating structure, cross-sectional observations of the samples were conducted in Figure 3. For Samples 1#–3#, due to the short sputtering duration for the metal coating, only discrete granular deposits were detected on the outer surfaces of the fiber filaments. In contrast, from Sample 4# onward, distinct continuous metal coatings became observable: the thickness of the metal coating on Sample 4# was approximately 70–80 nm, while that on Sample 5# ranged from 110 to 130 nm. Similarly, since the metal coating had not yet formed a continuous film in Samples 6#–8#, only a single-layer SiO_2_ film was observed, with a thickness of ~50–70 nm. For Samples 9# and 10#, a bilayer structure was clearly identified: the bottom layer corresponded to the metal coating, and the upper layer was the SiO_2_ coating.

**Figure 4:**
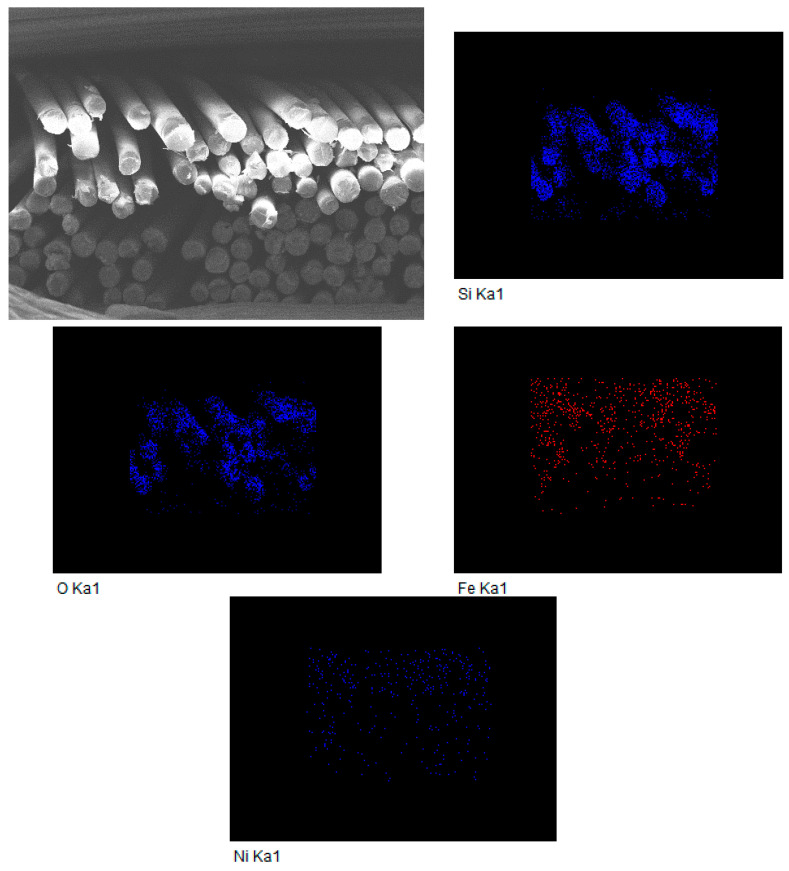
EDS results of 10# sample.

To further verify the Fe_70_Ni_30_/SiO_2_ bilayer architecture, energy-dispersive X-ray spectroscopy (EDS) was performed on the cross-sectional plane of Sample 10#, and the corresponding elemental maps for Fe, Ni, Si, and O are presented in Figure 4.

As shown in Figure 4, Si and O elements are distributed on the outer surface of the fiber, and the fiber’s outline is clearly visible, indicating that the outermost layer is SiO_2_. Additionally, the presence of Fe and Ni elements has been detected, confirming a dual-layer structure of FeNi/SiO_2_.

### 4.2. XRD Analysis for Structural Characterization

**Figure 5:**
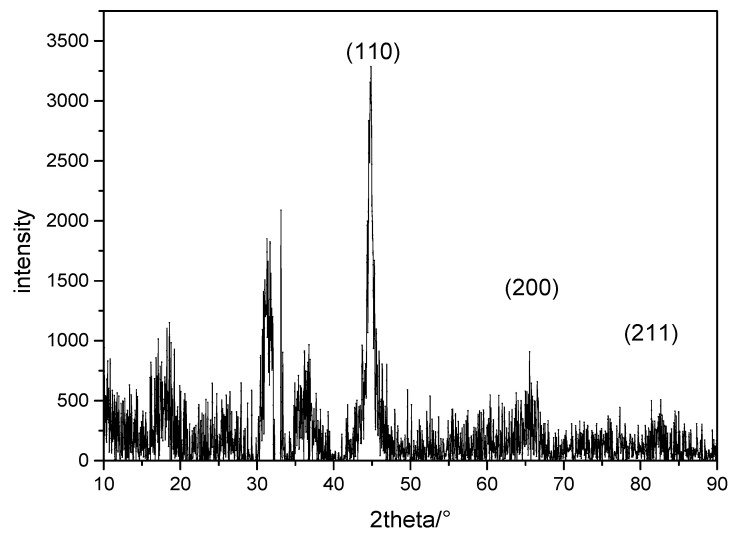
XRD Patterns of Sample 5#.

To characterize the chemical composition of the coatings, XRD measurements were performed on the samples. As revealed by the preceding SEM results, Sample 5# featured a relatively thick and continuous coating film; thus, it was selected for XRD analysis. The corresponding XRD pattern is presented in Figure 5.

Following peak extraction from the XRD data, the core diffraction peaks and corresponding phase identification results are summarized in the Table 2:

The Table 2 shows the core diffraction peaks at 44.74°, 65.56°, and 82.66° correspond to the (110), (200), and (211) planes of a body-centered cubic (bcc) structure. Using Cu Kα radiation (λ = 1.5406 Å), the interplanar spacings are calculated as d_110_ = 2.024 Å, d_200_ = 1.422 Å, and d_211_ = 1.166 Å. The average lattice constant a ≈ 2.86 Å, consistent with the bcc α-Fe(Ni) solid solution (PDF card 00-006-0696). The relative intensity ratio (110):(200):(211) = 100:29:16, derived directly from the measured diffraction pattern, deviates slightly from the standard random powder ratio, suggesting a modest preferred orientation in the sample. No extraneous peaks are detected, confirming a single-phase solid solution.

### 4.3. Electromagnetic Parameter Analysis

**Figure 6:**
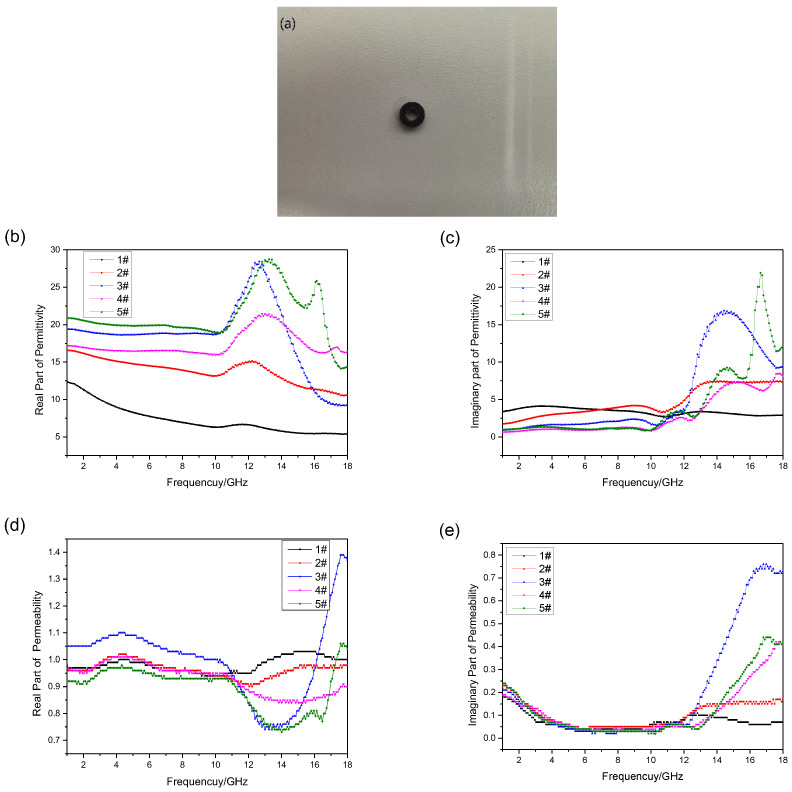
Electromagnetic Parameter Characterization Results. (**a**) Sample; (**b**) Real Part of Permittivity; (**c**) Imaginary Part of Permittivity; (**d**) Real Part of Permeability; (**e**) Imaginary Part of Permeability.

**Figure 7:**
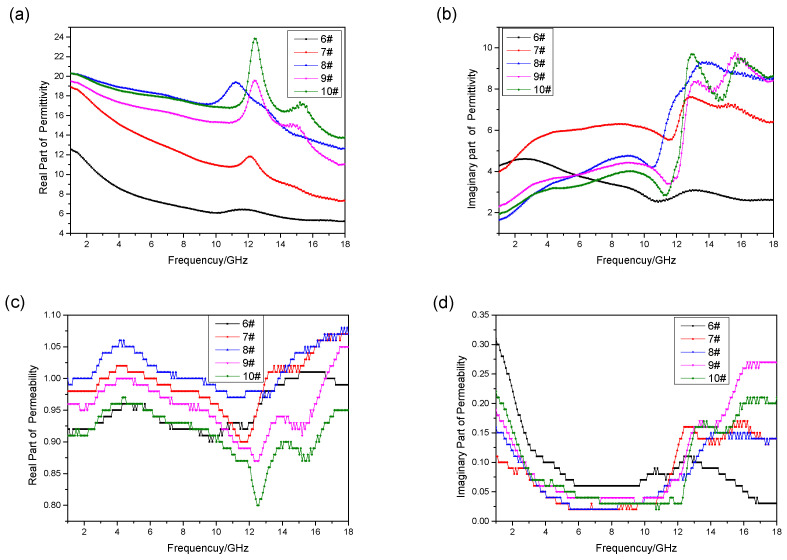
Electromagnetic Parameter Characterization Results. (**a**) Real Part of Permittivity; (**b**) Imaginary Part of Permittivity; (**c**) Real Part of Permeability; (**d**) Imaginary Part of Permeability.

Electromagnetic parameter characterization was performed using the coaxial transmission/reflection method. The as-prepared laminates were machined to a configuration with an outer diameter of Φ7 mm and an inner diameter of Φ3 mm; the test samples are illustrated in the figure below. The electromagnetic parameters of Samples 1# to 10# were characterized using the coaxial method, and the corresponding results are presented in the Figure 6 and Figure 7.

From the electromagnetic parameter characterization results, we can see that the composite exhibits predominantly dielectric loss behavior, while its complex permeability remains close to unity ($\mu^{\prime }$ ≈ 1.0, $\mu^{\prime \prime }$≪ 1) across the measured frequency range-confirming negligible magnetic loss contribution. The $\varepsilon^{\prime }$ of Samples 1# to 5# shows a non-monotonic variation (first increasing, then decreasing) with the extension of coating time. The 2 min sample has the lowest $\varepsilon^{\prime }$ (12.37 at 1.0 GHz), the 10 min sample reaches the peak $\varepsilon^{\prime }$ (19.42 at 1.0 GHz), and the 20 min sample sees $\varepsilon^{\prime }$ drop back to 20.92 (at 1.0 GHz). This trend is closely associated with the coating growth mechanism:

Initial stage (2–10 min): Prolonged coating time increases the coating thickness and surface coverage, which enhances the number of interfacial polarization sites between the fiber cloth and Fe_70_Ni_30_ alloy. The strengthened polarization capability thus leads to an increase in $\varepsilon^{\prime }$.

Later stage (10–30 min): Excessively long coating time causes grain agglomeration, increased surface roughness, and crack formation in local areas, which weakens the interfacial polarization effect. Meanwhile, the enhanced conductive loss from the excessive metal coating inhibits the polarization response, resulting in a decrease in $\varepsilon^{\prime }$.

For the dielectric loss factor $\varepsilon^{\prime \prime }$, it remains stable in the low-frequency range (1.0–10.0 GHz) but increases significantly in the high-frequency range (10.0–18.0 GHz). Notably, the longer the coating duration, the greater the increment of $\varepsilon^{\prime \prime }$ in the high-frequency range. At 18.0 GHz, $\varepsilon^{\prime \prime }$ of the 10 min sample reaches 8.20, while that of the 30 min sample rises to 12.21. This phenomenon stems from the multi-source contributions to dielectric loss:

① Conductive loss: prolonged coating time increases the coating thickness and free electron concentration, thereby enhancing conductive loss.

② Interfacial polarization loss: during 2–10 min, uniform coating growth increases interfacial polarization sites, boosting loss; during 10–30 min, despite weakened interfacial polarization, the dominant role of conductive loss drives a continuous rise in $\varepsilon^{\prime \prime }$.

③ Polarization relaxation loss: in the high-frequency range, grain refinement in the coating shifts the relaxation frequency toward higher frequencies, matching the test frequency band and leading to a significant enhancement of loss.

Samples 6# to 10# were prepared by depositing an additional SiO_2_ coating on the basis of Samples 1# to 5#. Analysis of the dielectric constant variation reveals that the real permittivity ($\varepsilon^{\prime }$) of both sample groups exhibits a non-monotonic trend (first increasing, then decreasing) as the Fe_70_Ni_30_ coating duration extends. However, the SiO_2_ coating significantly reduces $\varepsilon^{\prime }$ values across the entire frequency band, and the reduction magnitude is closely correlated with the Fe_70_Ni_30_ coating duration:

Low-frequency band (1.0 GHz): the $\varepsilon^{\prime }$ of Samples 6# to 10# decreases by 18.3–32.6% relative to the corresponding Samples 1# to 5#, with Sample 9# showing the largest reduction (32.6%) and Sample 6# the smallest (18.3%).

High-frequency band (18.0 GHz): the reduction ranges from 15.7% to 28.9%, with samples with medium-to-long Fe_70_Ni_30_ coating durations (8# and 9#) still exhibiting more pronounced reductions.

This phenomenon arises because SiO_2_ is a low-dielectric material ($\varepsilon^{\prime }$ ≈ 3.9): the deposited SiO_2_ layer forms a “low-dielectric shell”, which not only diminishes the overall polarization capability of the composite but also blocks direct contact between the Fe_70_Ni_30_ coating and air, reducing interfacial polarization sites. For samples with longer Fe_70_Ni_30_ coating durations, the original Fe_70_Ni_30_ coating has higher surface roughness and richer polarization sites, so the coverage effect of the SiO_2_ coating is more significant, leading to a larger reduction in $\varepsilon^{\prime }$. In contrast, for the short-coating-duration sample (6#), the Fe_70_Ni_30_ coating has low coverage and limited polarization contribution, resulting in a weaker regulatory effect of SiO_2_.

The effect of the SiO_2_ coating on the dielectric loss factor ($\varepsilon^{\prime \prime }$) exhibits a frequency-dependent behavior characterized by “low-frequency suppression and high-frequency synergistic enhancement”:

Low-frequency range (1.0–10.0 GHz): the $\varepsilon^{\prime \prime }$ of Samples 6# to 10# decreases by 8.7–23.5% relative to Samples 1# to 5#. Notably, the longer the Fe_70_Ni_30_ coating duration, the greater the reduction-with Sample 10# showing the maximum reduction of 23.5%.

High-frequency range (10.0–18.0 GHz): the $\varepsilon^{\prime \prime }$ of Samples 6# to 10# increases by 5.3–17.8% compared to the control group (Samples 1# to 5#), with Sample 8# (Fe_70_Ni_30_ coating for 10 min) exhibiting the most significant increase (17.8%).

The underlying mechanisms are as follows:

① Low-frequency range: As an insulating layer, the SiO_2_ coating inhibits the migration of free electrons in the Fe_70_Ni_30_ coating, reducing conductive loss. Additionally, at low frequencies, the interfacial polarization relaxation rate is well-matched to the electric field variation; the coverage of polarization sites by SiO_2_ weakens polarization loss. These two effects synergistically reduce $\varepsilon^{\prime \prime }$.

② High-frequency range: The SiO_2_ coating forms a new “Fe_70_Ni_30_/SiO_2_” interface with the Fe_70_Ni_30_ layer, introducing additional interfacial polarization sites. Meanwhile, at high frequencies, the polarization relaxation frequency matches the test frequency range, significantly enhancing interfacial polarization loss. This offsets the reduction in conductive loss, ultimately leading to an increase in $\varepsilon^{\prime \prime }$.

Notably, the Fe_70_Ni_30_ coating of the 10 min sample (Sample 8#) exhibits the highest uniformity, forming the most regular interface with SiO_2_. This results in the strongest interfacial polarization effect, hence the largest high-frequency increase in $\varepsilon^{\prime \prime }$.

### 4.4. Analysis of Microwave Absorption Performance

For microwave-absorbing materials, the reflection loss (RL) can be calculated using the following formula, and the software that we used is MATLAB R2018:

$$R=20\log_{10}\left|\frac{\sqrt{\frac{\mu^{\prime }-{j\mu }^{\prime \prime }}{\varepsilon^{\prime }-{j\varepsilon }^{\prime \prime }}}tanh\left(jdf\frac{2\pi }{c}\sqrt{{(\mu }^{\prime }-{j\mu }^{\prime \prime })(\varepsilon^{\prime }-{j\varepsilon }^{\prime \prime })}\right)-1}{\sqrt{\frac{\mu^{\prime }-{j\mu }^{\prime \prime }}{\varepsilon^{\prime }-{j\varepsilon }^{\prime \prime }}}tanh\left(jdf\frac{2\pi }{c}\sqrt{{(\mu }^{\prime }-{j\mu }^{\prime \prime })(\varepsilon^{\prime }-{j\varepsilon }^{\prime \prime })}\right)+1}\right|$$

**Figure 8:**
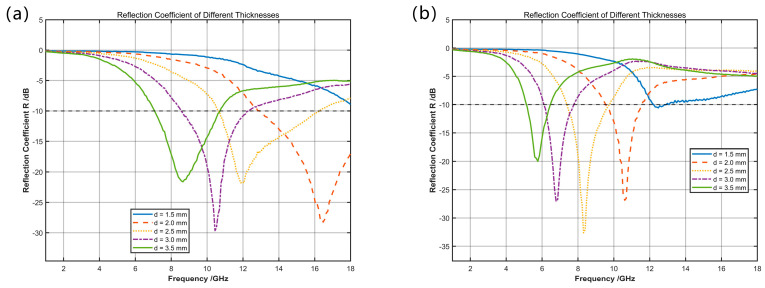

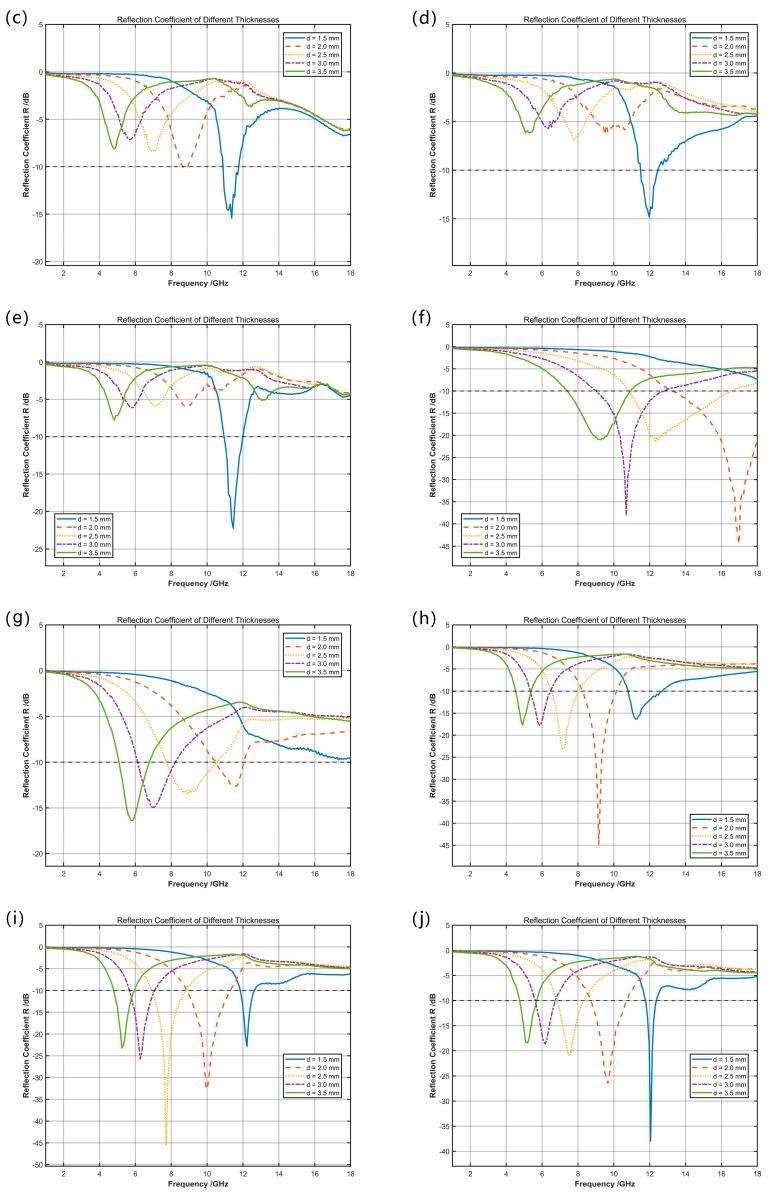
Simulation Results of Reflectance for Single-Layer Materials of Different Thicknesses: (**a**) 1#, (**b**) 2#, (**c**) 3#, (**d**) 4#, (**e**) 5#, (**f**) 6#, (**g**) 7#, (**h**) 8#, (**i**) 9#, (**j**) 10#.

Based on the aforementioned formula, simulation calculations were performed to evaluate the normal-incidence reflection loss (RL) performance of Samples 1# to 10# at varying thicknesses. The calculation results are presented in Figure 8:

The reflection loss (RL) curves of all samples exhibit a distinct thickness-dependent characteristic: the absorption peak position shifts toward lower frequencies with increasing thickness, while the absorption intensity (minimum RL value, R_min) first enhances and then weakens, which aligns with the “thickness-wavelength matching” principle in transmission line theory-specifically, as thickness increases from 1.5 mm to 3.5 mm, the absorption peak gradually moves from the high-frequency band (12–16 GHz) to the mid-frequency band (8–10 GHz) because increased thickness extends the propagation path of electromagnetic waves within the material, causing the phase matching condition to be satisfied at lower frequencies, demonstrating the precise regulation of absorption frequency bands by thickness, and the minimum RL value first decreases (absorption performance enhances) and then increases (absorption performance degrades) with increasing thickness, with most samples achieving optimal absorption intensity (R_min < −20 dB), some reaching −45 dB at thicknesses of 2.5–3.0 mm, as both overly thin and overly thick samples lead to poor absorption performance due to impedance mismatch.

For Samples 1#–5# without SiO_2_ coating, at 1.5 mm thickness, absorption peaks are concentrated in the high-frequency band (12–18 GHz) with a narrow EAB (<3 GHz) and R_min of ~−10 to −15 dB due to insufficient thickness causing impedance mismatch that prevents full electromagnetic wave attenuation; at 2.0–2.5 mm thickness, peaks shift to the mid-high frequency band with R_min significantly reduced to −20 to −30 dB, and notably, the sample with 5 min Fe_70_Ni_30_ coating achieves R_min = −33 dB at 2 mm, corresponding to optimal impedance matching between thickness and the electromagnetic parameters of the Fe_70_Ni_30_ coating; and at 3.0–3.5 mm thickness, peaks further shift to the mid-frequency band (8–10 GHz) with R_min rising to −10 to −15 dB and bandwidth narrowing due to excessive thickness causing multiple electromagnetic wave reflections and reducing energy attenuation efficiency.

In contrast, the introduction of the SiO_2_ coating in Samples 6#–10# improves the absorption performance of the composite in three key aspects: enhanced absorption intensity: as at the same thickness, R_min of SiO_2_-coated samples is 5–10 dB lower than that of uncoated samples, with R_min generally below −25 dB at 2.0–3.0 mm and the sample with 10 min Fe_70_Ni_30_ coating + SiO_2_ achieving R_min = −45 dB at 2 mm, a significant performance boost; broadened effective bandwidth: as the SiO_2_ coating introduces Fe_70_Ni_30_/SiO_2_ interfacial polarization, this optimizes impedance matching and broadens the EAB by 2–3 GHz, which stabilizes absorption performance, particularly in the mid-high frequency band (8–14 GHz); and improved thickness adaptability: as the optimal absorption thickness range is expanded from 2.5–3.0 mm to 2.0–3.0 mm, this reduces the sensitivity of absorption performance to thickness and provides greater flexibility for lightweight material design.

### 4.5. Optimization of Microwave Absorption Performance

The microwave absorption performance of the aforementioned single-layer Fe_70_Ni_30_/SiO_2_ composite-coated fabric absorber is constrained by its fixed thickness and intrinsic electromagnetic parameters. Its EAB spans only 3–5 GHz, failing to meet the wideband absorption requirements for practical engineering applications. This limitation arises from the coupled trade-off between impedance matching and energy attenuation in single-layer structures: when the thickness satisfies the quarter-wavelength matching condition for one frequency band, impedance mismatch occurs in other bands, leading to degraded absorption performance. For example, at 1.5 mm thickness, absorption peaks are confined to the high-frequency band (12–16 GHz), while at 3.5 mm, peaks shift to the mid-frequency band (8–10 GHz)—neither configuration achieves efficient broadband absorption.

To address the limitations of single-layer structures, a genetic algorithm (GA) is employed for the optimization design of multilayer composite structures. Through parametric encoding, fitness function construction, and iterative optimization, impedance gradient and loss synergy are achieved, thereby broadening the microwave absorption frequency band.

**Figure 9:**
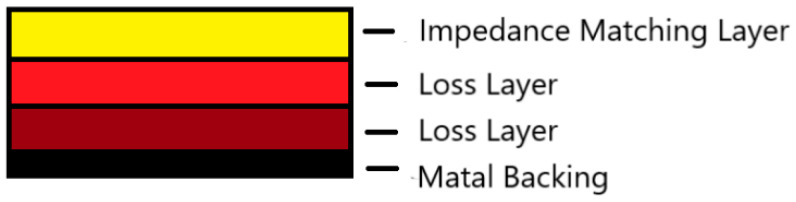
Schematic of the Multilayer Structure Design.

**Figure 10:**
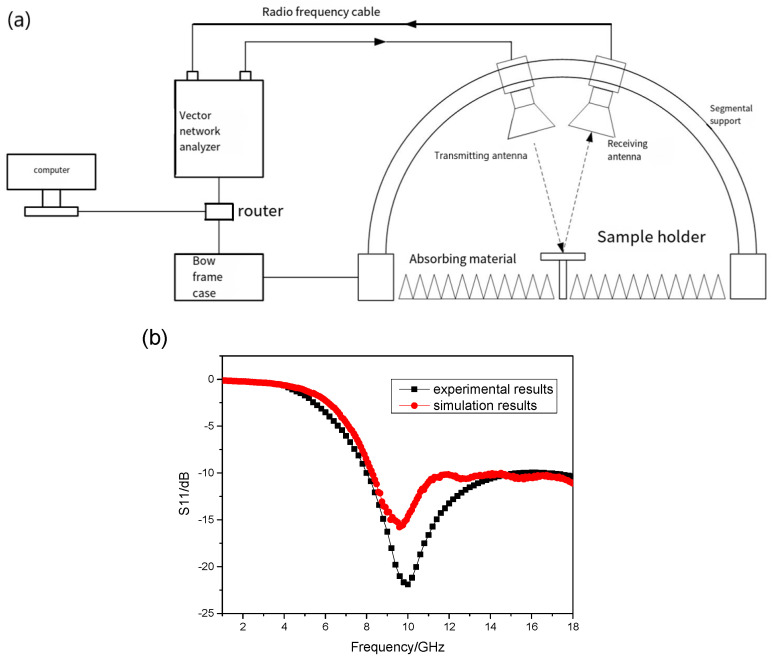
The experimental results (**a**) The arch method measurement system; (**b**) The comparison of experimental results and simulation results.

After calculation, a multilayer absorbing structure was obtained that can be realized within the range of 8 to 18 GHz at a thickness of 3.8 mm, as shown in Figure 9:

Herein, the impedance matching layer serves as a wave-transparent layer, which is an uncoated glass fiber-reinforced resin matrix composite (GFRP) with a thickness of 1.52 mm. The second layer is Sample 1# (0.76 mm thick), the third layer is Sample 6# (0.76 mm thick), and the bottom layer is a metal backplane. The total thickness of the structure is 3.8 mm. The electrical performance simulation results are presented in Figure 10b.

From the surface layer to the metal backplane, the gradient variation of the material’s electromagnetic parameters enables smooth impedance matching between the input impedance and free-space impedance, thereby minimizing interface reflections. The dielectric loss within each layer dominates in distinct frequency bands, forming a broadband energy attenuation network. The synergistic interaction between the thickness and electromagnetic parameters of each layer broadens the EAB via interference effects.

To validate the simulation results of the normal-incidence reflectivity of the material, we fabricated samples with dimensions of 180 mm × 180 mm. The reflectivity was measured using the arch method, and the experimental results are presented in the following figure:

The measured results presented in Figure 10 exhibit a good consistency with the simulation results. Both demonstrate an absorption of more than −10 dB across the frequency range of 8–18 GHz, with the absorption peaks centered at approximately 10 GHz. The variation in absorption depth stems from the thickness errors of each material layer. Given that the single-layer thickness of the prepreg is 0.1 mm, it is not feasible to achieve a precise thickness of 0.76 mm, which results in a slight shift in the absorption peak and a change in its maximum absorption depth.

Moreover, a comparative analysis of the recent literature on FeNi alloys as microwave-absorbing materials was conducted, systematically evaluating four key parameters: fabrication methodology, sample thickness, maximum reflection loss (RL), and effective absorption bandwidth (EAB, defined as the frequency range where RL ≤ −10 dB). As you can see in Table 3.

Compared with the current state of the art, this work offers three distinctive advances: (1) the magnetron sputtering deposition of a Fe_70_Ni_30_ alloy layer onto a continuous glass fiber-reinforced polymer composite substrate-thereby integrating broadband microwave absorption with structural load-bearing functionality, a dual-functional integration seldom reported for FeNi-based absorbers; (2) the design and realization of a three-layer impedance-gradient architecture optimized via a genetic algorithm, achieving an effective absorption bandwidth (EAB, RL ≤ −10 dB) of 10 GHz (8–18 GHz) at a total thickness of merely 3.8 mm-among the thinnest and broadest-performing configurations reported to date for FeNi-based multilayer absorbers; and (3) the incorporation of a conformal SiO_2_ surface coating, which enables precise modulation of dielectric properties and thereby significantly enhances impedance matching across the target frequency band.

## 5. Conclusions

This study fabricated Fe_70_Ni_30_/SiO_2_ bilayer-coated continuous glass fiber composites via magnetron sputtering and SiO_2_ encapsulation, systematically investigating the effects of Fe_70_Ni_30_ deposition duration and SiO_2_ modification on electromagnetic parameters and microwave absorption (MA) performance, and optimizing the multilayer structure by genetic algorithm (GA) for broadband absorption. Key findings are as follows:

1. Fe_70_Ni_30_ coating evolves from dispersed particles to a continuous film with extended deposition duration, and 30 min deposition yields the most uniform coating (Fe_70_Ni_30_ solid solution) with a well-defined bilayer structure after SiO_2_ encapsulation.

2. SiO_2_ coating significantly modulates dielectric parameters but barely affects magnetic properties of Fe_70_Ni_30_: it reduces the real dielectric constant ($\varepsilon^{\prime }$) by 15.7–32.6% across the whole frequency band, suppresses low-frequency (1.0–10.0 GHz) dielectric loss ($\varepsilon^{\prime \prime }$) by 8.7–23.5% and enhances high-frequency (10.0–18.0 GHz) $\varepsilon^{\prime \prime }$ by 5.3–17.8%. This is attributed to SiO_2_’s low dielectricity (inhibiting free electron migration) and the Fe_70_Ni_30_/SiO_2_ interface effect (increasing high-frequency polarization sites), with the 30 min Fe_70_Ni_30_ sample showing the strongest interface polarization.

3. Material thickness regulates MA performance (MA peak red-shifts with thicker thickness, RL_min_ first drops and then rises), with the optimal single-layer thickness of 2.5–3.0 mm. SiO_2_ coating further optimizes MA performance (RL_min_ reduced by 5–10 dB, EAB broadened by 2–3 GHz, optimal thickness expanded to 2.0–3.0 mm).

4. GA-optimized 3.8 mm three-layer composite realizes impedance gradient and loss synergy, expanding EAB from 4.8 GHz (single-layer) to 10 GHz (8.0–18.0 GHz) for broadband absorption.

In conclusion, this study realizes precise regulation of the MA performance of Fe_70_Ni_30_/SiO_2_-coated continuous glass fiber composites through process parameter optimization and structural design, providing experimental and technical support for the development of lightweight, high-efficiency and broadband microwave-absorbing materials.

## Figures and Tables

**Table 1:** Summary of Process Parameter Variations.

Sample	Power Supply	Target Material	Power (kW)	Sputtering Time (min)
1#	DC	Fe_70_Ni_30_	0.5	2
2#	DC	Fe_70_Ni_30_	0.5	5
3#	DC	Fe_70_Ni_30_	0.5	10
4#	DC	Fe_70_Ni_30_	0.5	20
5#	DC	Fe_70_Ni_30_	0.5	30
6#	DC + RF	Fe_70_Ni_30_ + SiO_2_	0.5 + 0.2	2 min + 10 min
7#	DC + RF	Fe_70_Ni_30_ + SiO_2_	0.5 + 0.2	5 min + 10 min
8#	DC + RF	Fe_70_Ni_30_ + SiO_2_	0.5 + 0.2	10 min + 10 min
9#	DC + RF	Fe_70_Ni_30_ + SiO_2_	0.5 + 0.2	20 min + 10 min
10#	DC + RF	Fe_70_Ni_30_ + SiO_2_	0.5 + 0.2	30 min + 10 min

**Table 2:** Core Diffraction Peaks and Corresponding Phase Identification Results.

Diffraction Angle2θ (°)	Interplanar Spacing d (Å)	Relative Intensity (%)	Matched Phase	Standard PDF Card	Corresponding Crystal Plane (hkl)
~44.74	~2.024	100	Body-centered cubic (bcc) Fe-Ni solid solution (α-phase)	00-006-0696 (α-Fe)	(110)
~65.56	~1.422	29			(200)
~82.66	~1.166	16			(211)

**Table 3:** Comparison of key electromagnetic wave absorption performance from the recent literature.

No.	Material System	Preparation Method	Thickness (mm)	RL_min (dB)	EAB (GHz)
1	Porous FeNi alloys [23]	Spray pyrolysis–reduction	1.80	−60.20	3.38
2	FeNi_3_@C core–shell [24]	In situ polymerization + thermal carbonization	2.13	−53.94	5.0
3	FeNi@C core–shell [25]	Pyrolytic carbon coating	1.70	−54.65	5.60
4	FeNi_3_/CNTs@SiO_2_ [26]	Core–shell + 3D network	2.2	−54.37	7.12
5	Multilayer FeNi_3_@C (ultra-thin broadband + superstructure full-band) [27]	Hierarchical interface engineering	1.6/9.8	—	7.4/16
6	FeNi alloy/graphene foam (FNGF) [28]	Self-assembly hydrothermal	2.09	−63.76	6.24
7	CMF/(FeNi)_x_(SiO_2_)_1−x_ nanoparticle film/carbon foam [22]	Magnetron sputtering	2.7	−56.3	8.0
8	Hollow FeNi@SiO_2_@PPy nanorods [21]	Hydrothermal + ion exchange	1.73	−76.38	5.42
9	Si_3_N_4_/Graphene bilayer (GA-optimized) [29]	CVD + genetic algorithm	5.0	—	12.481
10	TCFF two-layer periodic carbon fiber fabric (GA-assisted optimization) [30]	Interface engineering + electrodeposition	6.0	—	14.79 (4–18 GHz)
11	E-glass fiber aerogel-like composites [31]	3D hollow glass fiber embedding	—	—	11.65
This work	Fe_70_Ni_30_/SiO_2_/E-glass fiber	Magnetron sputtering	3.8	−21.9	10.0 (8–18)

## Data Availability

The original contributions presented in this study are included in the article. Further inquiries can be directed to the corresponding author.
